# Acoustic Indoor Localization System Integrating TDMA+FDMA Transmission Scheme and Positioning Correction Technique

**DOI:** 10.3390/s19102353

**Published:** 2019-05-22

**Authors:** Xiang Chen, Yuheng Chen, Shuai Cao, Lei Zhang, Xu Zhang, Xun Chen

**Affiliations:** Department of Electronic Science and Technology, University of Science and Technology of China, Hefei 230027, China; cyh1102@mail.ustc.edu.cn (Y.C.); caoshuai@ustc.edu.cn (S.C.); zl2017@mail.ustc.edu.cn (L.Z.); xuzhang90@ustc.edu.cn (X.Z.); xunchen@ustc.edu.cn (X.C.)

**Keywords:** Indoor localization, Chirp signal, TDOA, TDMA+FDMA, Doppler shifts

## Abstract

This paper presents a novel audio indoor localization system. In the proposed system, four speakers placed at known positions transmit chirp signals according to the time-division multiple access (TDMA) plus frequency-division multiple access (FDMA) transmission scheme. A smartphone receives the signal via a built-in microphone and calculates the time differences of arrival (TDOAs). Using TDOA measurements, the position is estimated by the shrinking-circle method. In particular, to reduce the positioning error in moving conditions, a TDOA correction method based on Doppler shifts is proposed. The performance of the proposed system was evaluated in real-world experiments using a 10.971 m × 5.684 m positioning area. The results of the static-target positioning experiment showed that the TDMA+FDMA transmission scheme has more advantages in improving the update rate of the positioning system than the TDMA-only transmission scheme. The results of the moving-target positioning experiment under three different speeds demonstrated that the positioning errors were reduced by about 10 cm when the Doppler-shift-based TDOA correction method was adopted. This research provides a possible framework for the realization of a TDOA-chirp-based acoustic indoor positioning system with high positioning accuracy and update rate.

## 1. Introduction

With the development of intelligent information processing technology and smartphones, location-based services (LBS) have attracted increased attention. The key for LBS is seamless indoor and outdoor navigation for users. Although a global positioning system (GPS) or Beidou can work well for outdoor positioning, they are not effective solutions for indoor positioning due to the blockage by buildings. Bluetooth, Ultra Wideband (UWB), ultrasound, WiFi, etc., have been used successfully to realize indoor positioning in many systems. The cost of UWB-based systems is relatively high, and smartphone sensors often cannot meet the bandwidth required by the system [1,2]. Ultrasound-based systems also cannot be implemented on smartphones because the signal frequency band often is outside the acceptance range of the mobile phone microphone [3,4,5,6,7]. Although WiFi and Bluetooth-based systems are easy to set up due to the wide application of WiFi and Bluetooth, the positioning accuracy of both these systems can only reach the meter level [2,8], which cannot satisfy the needs of high-precision indoor positioning.

Recently, audio-based indoor positioning technology that operates by emitting and receiving acoustic signals between nodes and the positioning target has attracted increased attention due to its unique advantages. Related research shows that audio-based indoor positioning systems can achieve decimeter-level accuracy [9,10,11]. In addition, audio-based technology is a low-cost indoor positioning solution because every commercial off-the-shelf (COTS) smartphone has a built-in speaker and microphone, and many buildings or public places—such as hospitals, shopping malls, airports, etc.—have their own speaker and microphone systems. 

In audio-based indoor positioning systems, code-division multiple access (CDMA) [12,13,14] and linear chirp are the two common audio coding methods [9,10,11,15,16]. Fernando J. Álvarez et al. realized an audio-based indoor positioning system that utilized CDMA signals [12]. They evaluated the system performance under different signal-to-noise ratios (SNRs) by setting two criteria, system availability (SA, defined as the percentage of measurements whose error is below the outlier’s threshold) and mean positioning error (MPE). At the center of the positioning area, the SA was 90%, and the MPE was 8 cm when the SNR was 12 dB. Fabian Hoflinger et al. realized an audio-based indoor positioning system that utilized chirp signals, and the MPEs were observed to be less than 30 cm for static points and 34 cm for smartphone tracking [9]. Although the positioning for static targets can be highly accurate most of the time, many problems appear when the positioning target is moving. Sérgio I.Lopes analyzed the ambiguity functions of CDMA signal and chirp signal with the same frequency band (18–22 kHz) and time duration and found that the CDMA signal was easily influenced by Doppler shifts when the target was moving at a high speed [10,17]. Compared to CDMA, chirp signal pulses have a Doppler tolerance of up to ±bandwidth/10. That is to say, chirp-based systems may be more accurate for moving target positioning than CDMA-based systems. 

Regarding the aspect of acoustic signal measurement, measurements including the angle of arrival (AOA), the time difference of arrival (TDOA), the time of arrival (TOA), and the frequency difference of arrival (FDOA) have been applied to realize audio-based indoor positioning in related research [18,19,20,21,22]. Among these methods, the measurement of TDOA has the advantage of not requiring clock synchronization between the target and the nodes. The FDOA method, which measures the frequency difference, can only be applied when there is relative motion between the target and the nodes. The joint usage of TDOA and FDOA can estimate the target position and the velocity accurately when the target is moving [23], and has attracted a lot of research interest in the fields of surveillance, navigation, wireless communications, and sensor networks [24,25,26,27].

Recently, to explore decimeter-level accuracy indoor positioning technology, our research group tried to develop an audio-based system by adopting TDOA measurement and chirp signals [28]. In the process of system implementation, some main factors were found to affect the system performance. As suggested in references, the detection performance of chirp signals was influenced by the time-bandwidth product (TBP), which is defined as the product of the duration of the signal and the bandwidth. Generally, the larger the TBP is, the higher the time resolution and the accuracy of chirp signal detection are. In related research [29], 18–22 kHz has been proven to be a suitable frequency band for indoor positioning, because it can make full use of the audio-signal-receiving ability of smartphones and can satisfy the insensitivity of human ears. Under the time-division multiple access (TDMA) transmission scheme, most chirp-based systems use the whole available bandwidth to generate chirp signals to increase the TBP [10]. However, using TDMA alone can result in some other problems. For instance, TDOA values calculated from at least three nodes are usually needed for location algorithms. At each time point, only a signal from one node can be received when the TDMA transmission scheme is adopted. Consequently, the TDOA values used for positioning are collected at different positions when the target is moving, which will inevitably bring about a positioning error. The other problem of TDMA is the low update rate, because the system starts positioning calculation only after receiving signals from all the nodes. 

To improve the positioning update rate and reduce the positioning error caused by target motion, a novel indoor positioning system based on chirp audio signals and TDOA was implemented in this study. The main features of this system can be summarized as follows: (1) a TDMA+FDMA (frequency-division multiple access) signal transmission scheme was adopted to improve the positioning update rate; (2) a target-moving-speed-based TDOA correction method was used to improve the positioning accuracy. The rest of this paper is organized as follows: Section 2 presents the overview and the detailed description of each part of the system; Section 3 presents location results and analysis for static targets and moving targets; and Section 4 presents a discussion and conclusions.

## 2. Methods

### 2.1. Overview of the TDOA-Chirp-Based System

**Figure 1:**
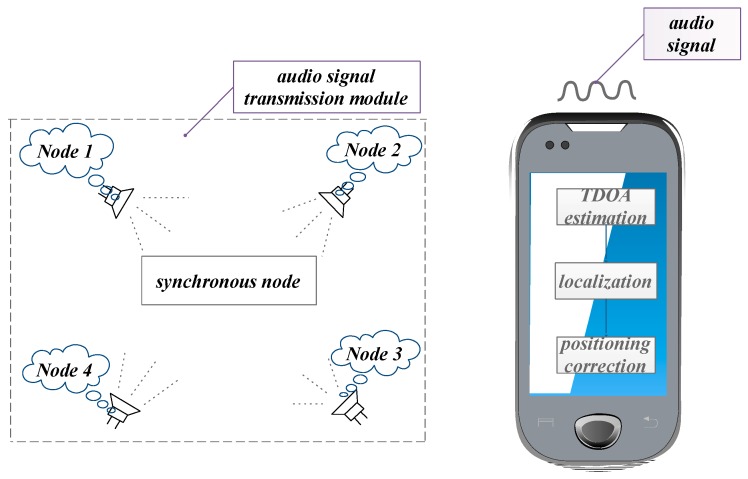
Diagram of the time difference of arrival (TDOA)-chirp-based system.

Figure 1 shows the diagram of the proposed TDOA-chirp-based indoor positioning system. The system mainly consisted of four modules, namely, the audio signal transmission module, the TDOA estimation module, the localization module, and the positioning correction module. 

**Figure 2:**
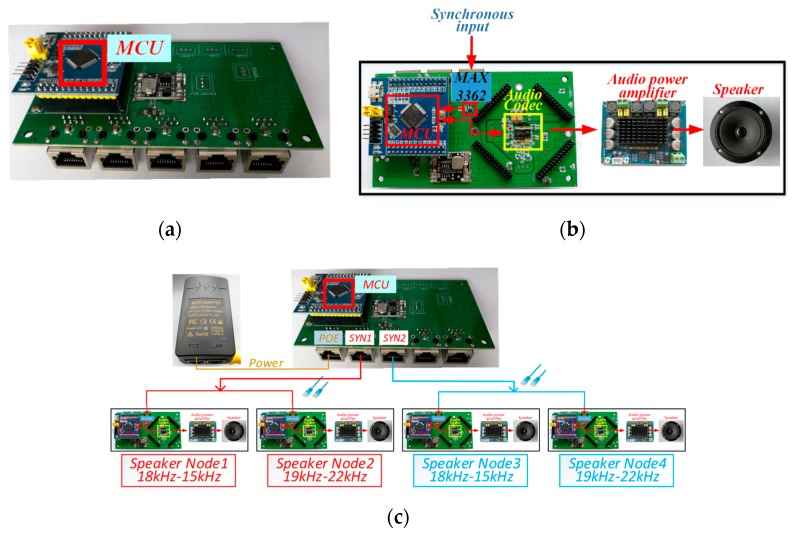
Diagram of the system hardware. (**a**) Synchronous node; (**b**) speaker node; (**c**) diagram of node connection.

The audio signal transmission module consisted of a synchronous node, which was used to generate trigger signals and four speaker nodes (Node1–4). The hardware structure of the synchronous node is shown in Figure 2a. The trigger signals were generated by a microcontroller unit (MCU) and converted by the transceiver (MAX3362) to a differential signal that controlled the transmission order of Node1–4. The hardware structure of the speaker node is shown in Figure 2b. Each node consisted mainly of four parts: an MCU, an audio codec, an audio power amplifier, and an omnidirectional speaker. After receiving the synchronization trigger signal, the MCU controlled the audio codec (TLV320AIC23B) to convert the digital audio signal into an analog signal. Then, the analog signal was amplified by an audio power amplifier and drove the speaker. The amplification factor of the audio power amplifier could be adjusted within a certain power range (0–50 W). The system was powered by a power over Ethernet (POE) module. The POE was used to convert 220V alternating current (AC) to 24V direct current (DC). Each node had a buck module to power the MCU, the audio codec, etc. Additionally, the synchronous node had two network ports to control the transmission order of the speaker node via a standard network cable, as shown in Figure 2c.

The TDOA estimation module was used to detect the arrival times of the chirp signals and calculate the TDOA. The localization module utilized the TDOA to give an initial estimated position. The positioning correction module corrected the initial estimated position to get more accurate positioning results. The detailed description of each module is given below. A positioning program consisting of a TDOA estimation module, a localization module, and a positioning correction module was written in C++ and was packaged into the App written in java. The whole App was installed in a smartphone (Huawei P10 Plus, Shanghai, China). The audio signal sampling frequency was 44.1 kHz.

### 2.2. FDMA+TDMA Audio Signal Transmission Scheme

**Figure 3:**
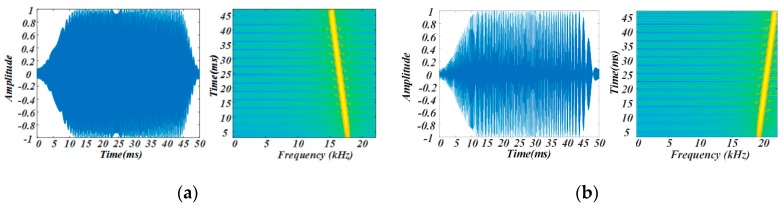
Waveform illustrations of chirp signals. (**a**) 18–15 kHz chirp signal; (**b**) 19–22 kHz chirp signal.

**Figure 4:**
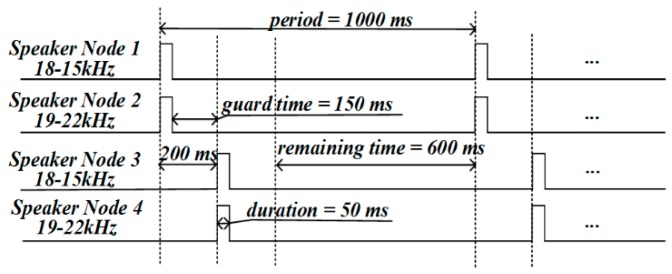
An example of time-division multiple access plus frequency-division multiple access (TDMA+FDMA) transmission scheme.

The frequency range of the audio chirp signal was set to 15–22 kHz in the proposed system. In FDMA, the 15–22 kHz frequency band was divided into two sub-bands to generate chirp signals. The frequency of the chirp signal shown in Figure 3a dropped from 18 to 15 kHz, and that of the chirp signal shown in Figure 3b rose from 19 to 22 kHz. In order to avoid the spectrum leakage caused by a sharp increase in amplitude of the chirp pulse, and to keep the pulse non-invasive, a combined window function (hamming window plus rectangular window) was used to modulate the amplitude of the chirp pulse. The 18-19 kHz protection bandwidth was used to avoid interference between the two chirp signals. In TDMA, the four speaker nodes were divided into two groups to transmit the two kinds of chirp signals. As shown in Figure 4, which gives an example of the TDMA+FDMA transmission scheme, Node 1 emitted 18–15 kHz chirp signal, and Node 2 emitted 19–22 kHz chirp signal synchronously; after 200 ms, Node 3 emitted 18-15 kHz chirp signal, and Node 4 emitted 19–22 kHz chirp signal synchronously. For a given positioning period, the signal duration, the guard time, and the remaining time needed to be designed. As shown in Figure 4, the positioning period was set to 1000 ms. Both the chirp signals had a 50 ms time duration. A 150 ms guard time was used to completely separate the two chirp signals, and a 600 ms remaining time was used to distinguish the positioning period. 

### 2.3. TDOA Estimation

When the microphone received audio signals, the TDOA estimation module could detect the arrival times of the chirp signals from different nodes. Generally, the arrival times of chirp signals were obtained as the lag time τ that maximized the cross correlation (CC) between the received signal and the emitted signal, as shown in Equation (1). $R_{pq}$ was the CC between the received signal, denoted as p[n], and the emitted signal, denoted as q[n]. E{·} stood for the mathematical expectation.

(1) $$\underset{m}{\tau =argmax}\left(R_{pq\left(m\right)}\right)\;R_{pq}\left[m\right]=E\{p\left[n\right]q\;\left[n+m\right]\}$$

However, in reality, the τ computed using Equation (1) was generally not the true time delay, since there existed a multipath effect. Large errors may have occurred when the maximum CC was used to determine the τ. Considering that the time of the direct path must have been earlier than τ, an alternative method was to use the time of a corresponding point whose value was greater than or equal to the product of a weight coefficient (0<α<1) and the maximum CC as the arrival time. α was determined according to the experimental environment.

(2) $$\{\begin{array}{c} TDOA_{ij}=t_{i}-t_{j}\;i\neq j;\;\left|i-j\right|\leq 1;\;and\;i,j\neq 2,3\\ TDOA_{ij}=t_{i}-t_{j}+200\;\;\;i\neq j;\;\left|i-j\right|\geq 1;\;and\;i,j=2,3 \end{array}$$

When $t_{i}$ and $t_{j}$ were used to represent the arrival times of the chirp signals the smartphone received from the *i*th node and the *j*th node, respectively, the $TDOA_{ij}$ between the two nodes could be calculated according to Equation (2).

### 2.4. Positioning Algorithm

The localization module was used to get the target position through the TDOA. When the $TDOA_{ij}$ was obtained, the distance difference between the *i*th and *j*th nodes could be calculated according to Equation (3).

(3) $$\{\begin{array}{c} d_{ij}=c\times TDOA_{ij}=\|\vec{x}-{\vec{x}}_{i}\|-\|\vec{x}-{\vec{x}}_{j}\|\\ c=331.45\times \sqrt{1+\frac{T}{273.15}} \end{array}$$

where $\vec{x}$ was the position to locate, ${\vec{x}}_{i}$ and ${\vec{x}}_{j}$ stood for the positions of the *i*th and the *j*th nodes, respectively, T was the temperature, and *c* represented the velocity of voice. The essence of localization using TDOA was an optimization problem, and many methods have been proposed to solve this problem, including the maximum-likelihood (ML) method [30], the non-linear least-squares (NLS) approach, the weighted least-squares (WLS) approach, the two-step weighted least-squares (2WLS) approach, the constrained weighted least-squares (CWLS) approach, the separated CWLS (SCWLS) approach [31,32,33,34], etc. 

**Figure 5:**
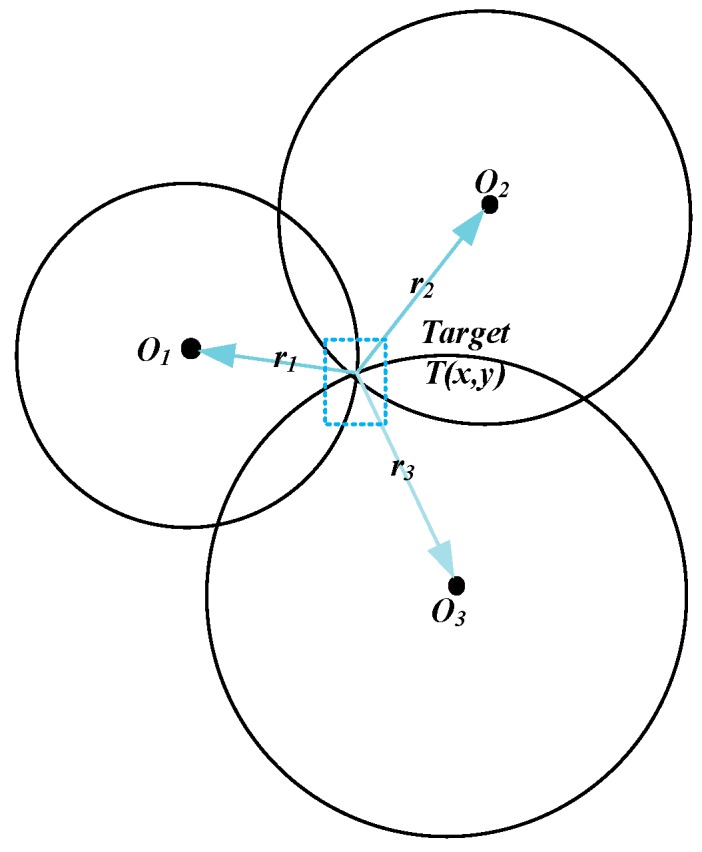
Basic idea of the shrinking-circle (SC) method.

In our system, the shrinking-circle (SC) method was used to realize target positioning due to its low complexity and high robustness [28]. As shown in Figure 5, $O_{i}\left(x_{i},y_{i}\right)$ denoted the coordinate of the *i*th node, T(x,y) denoted the target’s coordinate, $r_{i}$ represented the distance between the target and the ith node, the target T was on the circumference of a circle with $Q_{i}$ as the center and $r_{i}$ as the radius. Equation (4) depicts the relationship between these parameters. Taking the $1^{st}$ node as the reference, the radius of circle $O_{i}$ could be determined, as shown in Equation (5). As the $d_{i1}$ could be computed from the TDOA values, the radius $r_{1}$ was the only variable required to solve T(x,y). Consequently, the basic idea of the SC method is to find the perfect radius $r_{i}$ for which all the circles intersect at the same point. More details about the SC strategies can be found in our previous work [28].

(4) $${\left(x-x_{i}\right)}^{2}+{\left(y-y_{i}\right)}^{2}=r_{i}^{2}$$

(5) $$r_{i}=r_{1}+d_{i1}$$

### 2.5. Positioning Correction Considering Moving Speed of the Target

**Figure 6:**
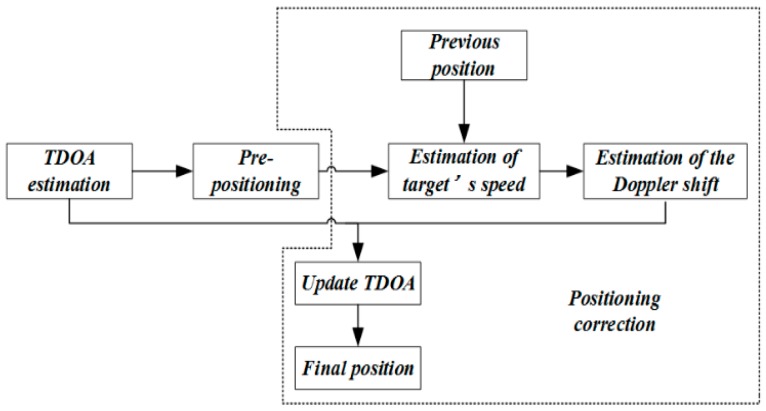
Diagram of positioning correction.

As mentioned above, the localization module utilized the TDOA to give an initial estimated position. However, the Doppler shift caused by target motion could influence TDOA estimation, resulting in a positioning error. Therefore, we proposed a positioning correction module to modify the initial estimated position and get more accurate positioning results. The details of the positioning correction are shown in Figure 6. 

In the positioning correction, it was indispensable to estimate the Doppler shift. The Doppler shift Δf caused by a moving target could be depicted using Equation (6) [35].

(6) $$\Delta f=\frac{f}{c}\times v\times cos\theta$$

where *f* was the carrier frequency, *c* was the velocity of voice, *v* was the speed of target, and θ stood for the angle between the directions of target motion and audio signal propagation. Supposing that *f* = 18 kHz, *v* = 1 m/s and $\theta =0^{{}^{\circ}}$, 52.9 Hz Doppler shift could be calculated. 

**Figure 7:**
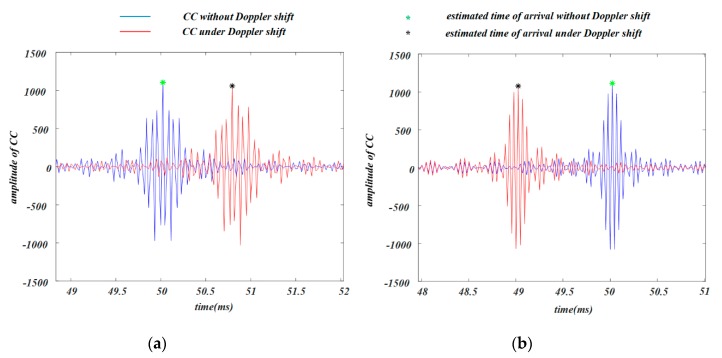
Simulation results of influences of Doppler shift on arrival time detection. (**a**) 18–15 kHz chirp signal; (**b**) 19–22 kHz chirp signal.

To analyze the effect of the Doppler shift, we conducted simulations under the following two situations: (1) the emitted signal was 18–15 kHz chirp signal, where one received signal was 18–15 kHz chirp signal with 50 ms time delay, and the other received signal was 18–15 kHz chirp signal with 52.9 Hz Doppler shift and 50 ms time delay; (2) the emitted signal was 19–22 kHz chirp signal, where one received signal was 19–22 kHz chirp signal with 50 ms time delay, and the other received signal was 19–22 kHz chirp signal with 52.9 Hz Doppler shift and 50 ms time delay. With weight coefficient α=1, the CC between the emitted and the received signals and the estimated arrival times were computed. As shown in Figure 7, the arrival time for the 18–15 kHz chirp signal with Doppler shift was about 50.8 ms, whereas the arrival time for the 19–22 kHz chirp signal with Doppler shift was about 49 ms. The simulation results show that the 52.9 Hz Doppler shift could cause about 1 ms error in arrival time estimation. Consequently, when the two kinds of chirp signals with Doppler shift were the received signals corresponding to two nodes, there existed about 2 ms TDOA estimation error, which could result in about 68 cm distance difference.

In this study, an effective method that considered the moving speed of the target was used to correct the positioning error caused by the Doppler shift. The basic idea was to convert the Doppler shifts in the frequency domain into time differences to update the TDOA values. To simplify the process, the linear chirp signal with Doppler shift was still supposed to be a linear chirp signal. The Doppler shift Δf was converted into the time difference Δt using Equation (7), where F was the frequency range of the chirp signals, and $T_{chirp}$ stood for the duration of the chirp signal. Since the Doppler shift could cause a delay in arrival of the 18–15 kHz chirp signal and advance the arrival of the 19–22 kHz chirp signal, the $TDOA_{ij}$ between the *i*th and the *j*th nodes was updated according to Equation (8).

(7) $$\Delta t=\frac{\Delta f}{F}\times T_{chirp}$$

(8) $$\{\begin{array}{c} {\vec{t}}_{i}=t_{i}-\Delta t\;\;\;i=1,3\\ {\vec{t}}_{i}=t_{i}+\Delta t\;\;i=2,4\\ TDOA_{ij\_new}={\vec{t}}_{i}-{\vec{t}}_{j} \end{array}$$

Based on the above introduction, to achieve the correction of the TDOA value, calculation of the Doppler shift was very critical. According to Equation (6), the Doppler shift was determined by *v* and θ. Assuming the current position and the previous position were denoted as $\hat{P}$ and $P_{prev}$, respectively, the moving speed *v* of the target could be computed using Equation (9), where $t^{\prime }$ was the time interval between $\hat{P}$ and $P_{prev}$.

(9) $$\{\begin{array}{c} \vec{v}=\frac{\hat{P}-P_{prev}}{t^{\prime }}\\ v=\|\vec{v}\| \end{array}$$

θ could be calculated using two vectors, as shown in Equation (11). The vector $\vec{v}$ is shown in Equation (9) and the vector ${\vec{d}}_{propagation}$ is shown in Equation (10), where $P_{i}$ was the position of the *i*th speaker node. According to Equations (7)–(11), Doppler disturbances were compensated by using the location and the speed of the mobile target estimated from the pre-positioning result before correction. However, large positioning errors caused by reverberation, outliers in the propagation times and occlusions, etc., may have caused large speed estimation errors. Therefore, a corresponding strategy should have been formulated to abandon the speed estimation results of obvious anomalies. Taking the indoor navigation of human walking as an example, in normal circumstances, the walking speed of a person is under 1.5 m/s. Therefore, 2 m/s could be set as a speed threshold. When the speed estimated by Equation (9) was above 2 m/s, it was considered to be inappropriate for Doppler shift calculation.

(10) $${\vec{d}}_{propagation}=\frac{\hat{P}-P_{i}}{\left\|\hat{P}-P_{i}\right\|}$$

(11) $$\theta =acos\left(\frac{\vec{v}\cdot {\vec{d}}_{propagation}}{\left\|\vec{v}\|*\|{\vec{d}}_{propagation}\right\|}\right)$$

### 2.6. Parameters for System Performance Evaluation

The localization error (*LE*) defined in Equation (12) and the cumulative distribution function (CDF) of the *LE* erre used to evaluate the system positioning performance in static positioning experiments. In Equation (12), *x* denoted the true position, and $\hat{x}$ was the estimate of the true position. In moving-target positioning experiments, the positioning error of each positioning, denoted as the moving-target localization error (*MTLE*), was defined as the shortest distance between the estimated positions and the track. The mean squared error (*MSE*) was used to evaluate the positioning performance in moving-target positioning experiments, as shown in Equation (13), where *N* was the number of estimated positions and $x_{i}$ was the estimated position.

(12) $$LE=\|\hat{x}-x\|$$

(13) $$MSE=\frac{1}{N}\overset{N}{\underset{i=1}{\sum }}MTLE\left(x_{i}\right)$$

## 3. Results

**Figure 8:**
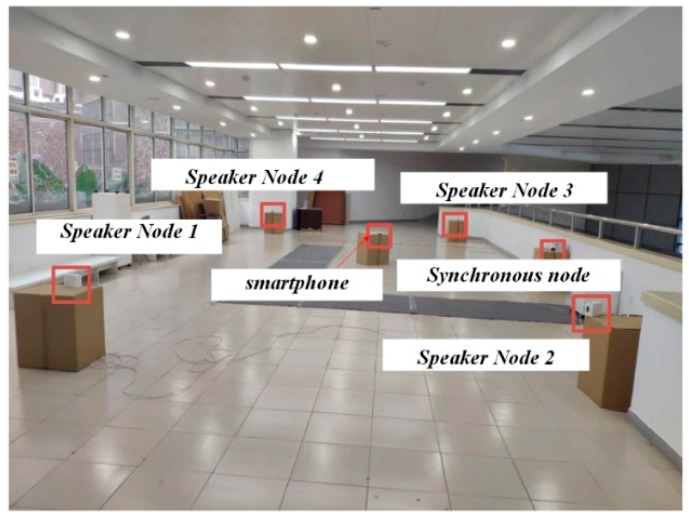
Photo of experimental setup.

The TDOA-chirp-based system was placed in a hall, as shown in Figure 8. The four speakers (Node 1 to Node 4) were placed at (0, 0), (0, 5.684), (10.971, 5.684), and (10.971, 0), respectively, to form a positioning area with a size of 10.971 m × 5.684 m. The synchronous node was set to control the speakers to emit chirp signals according to the TDMA+FDMA or the TDMA transmission schemes. Considering the effect of temperature on the speed of sound, the positioning experiment was generally carried out at the same temperature, around 15 °C. In addition, there was no other equipment that may have caused temperature inconsistencies in the experimental area. The smartphone collected audio signals through the built-in microphone. At the same time of collecting the chirp signals, a lot of noise—including talking sounds, footsteps, and ringtones of other smartphones—was also being collected. Since the noises were usually low-frequency noises, the received audio signal was preprocessed first by a finite impulse response (FIR) band-pass filter (200-order, 15–22 kHz) to reduce the influence of the noise. The SNR before filtering was 15 dB–20 dB, while it was 45 dB–50 dB after filtering. Considering the experimental environment, the value of α in TDOA estimation was set as 0.2. 

### 3.1. Signal Preprocessing and TDOA Estimation 

**Figure 9:**
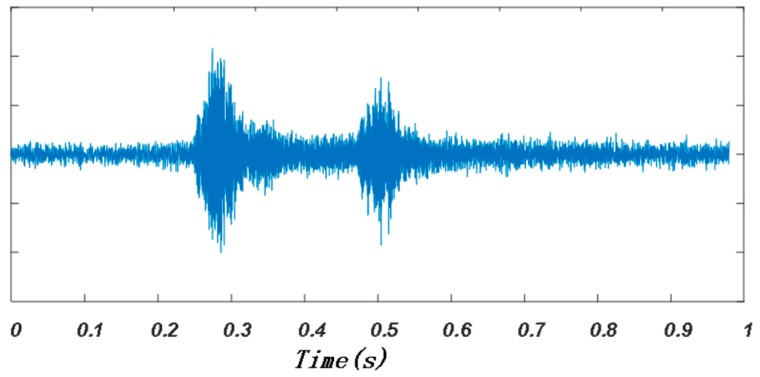
An example of the received audio signal during one positioning period.

**Figure 10:**
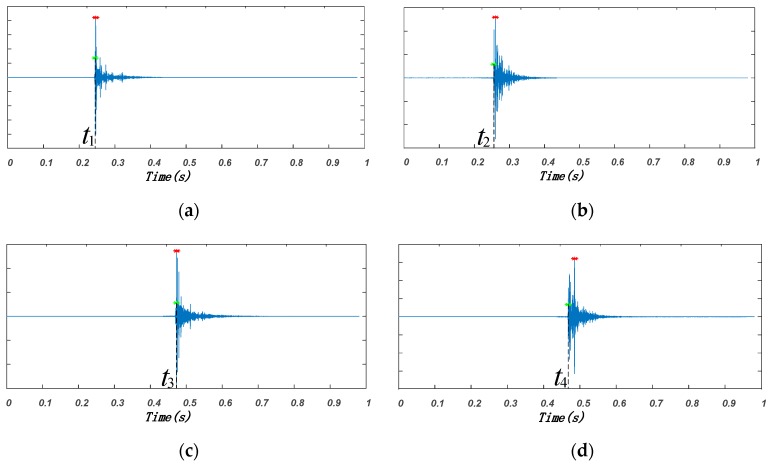
Estimation of arrival times for different nodes. (**a**) Cross correlation (CC) of Node 1; (**b**) CC of Node 2; (**c**) CC of Node 3; (**d**) CC of Node 4.

An example of the audio signal collected by the smartphone during one positioning period is shown in Figure 9. After filtering, the arrival time of the signal transmitted by each speaker node was estimated, as shown in Figure 10, in which the red point corresponded to the maximum of CC and the green point was the first point greater than 0.2 times the maximum. The time corresponding to the green point, which was denoted as $t_{1},t_{2},t_{3},t_{4}$, respectively $\left(t_{1}=0.2527s,t_{2}=0.2638s,t_{3}=0.4842s,t_{4}=0.4789s\right)$, was considered as the signal arrival time. According to Equation (2), the TDOA of any two speaker nodes could be calculated ($TDOA_{12}=-0.0111s$, $TDOA_{13}=-0.0315s$, $TDOA_{14}=-0.0262s$). 

### 3.2. Static-Target Positioning Experiments under Different Update Rates

To demonstrate the positioning characteristics of the proposed FDMA+TDMA scheme under high update rate, static-target positioning experiments with three different positioning periods were conducted for both TDMA+FDMA and TDMA-only schemes. 

As shown in Figure 4, the specific transmission scheme of TDMA+FDMA was related to parameters such as the period, the signal duration, the guard time, and the remaining time. As mentioned above, the accuracy of TDOA estimation was related to the chirp signal TBP. In the TDMA+FDMA transmission scheme, the chirp signals had a narrow frequency band. Hence, in the process of shortening the positioning period, the signal duration and the guard time should have remained unchanged for effective TDOA estimation and signal separation. In other words, the positioning period could be shortened only by shortening the remaining time. The specific parameter values of the three TDMA+FDMA transmission schemes are described in Table 1, where Scheme 1, Scheme 2, and Scheme 3 represent 1000 ms, 600 ms, and 450 ms positioning periods, respectively.

**Figure 11:**
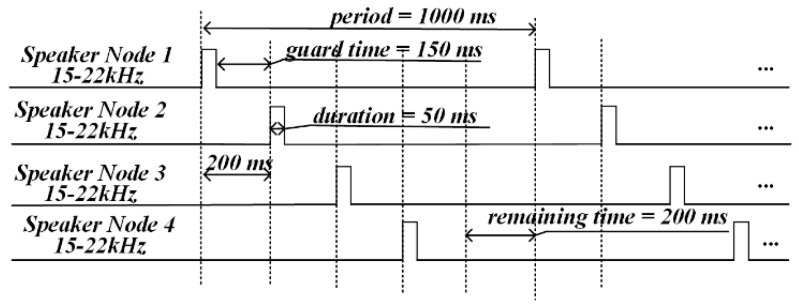
TDMA-only transmission scheme.

In the TDMA-only transmission scheme, the frequency band of the chirp signal was about twice that in the TDMA+FDMA transmission scheme. Hence, the positioning period could be shortened by reducing the signal duration, the guard time, and the remaining time. Taking the 1000 ms positioning period as an example, the TDMA-only transmission scheme is shown in Figure 11. Within the 1000 ms positioning period, the speakers Node 1, Node 2, Node 3, and Node 4 emitted 50 ms 15–22 kHz chirp signals in turn at 200 ms intervals. The specific parameter values of the three TDMA-only transmission schemes are described in Table 2, where Scheme 1, Scheme 2, and Scheme 3 represent 1000 ms, 600 ms, and 450 ms positioning periods, respectively.

**Figure 12:**
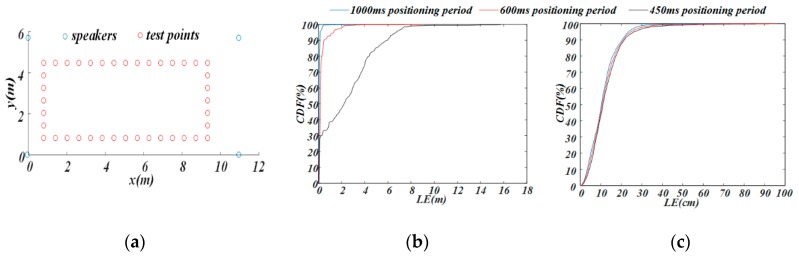
Results of static-target positioning experiment. (**a**) Test points; (**b**) TDMA-only scheme; (**c**) TDMA+FDMA scheme.

In the positioning area, 40 test points (marked in red in Figure 12a) were selected. During the experiment, the smartphone was placed on the test points to finish the positioning. Approximately 90 positioning results were collected at each point. Figure 12b gives the CDF of the *LE* under TDMA-only transmission schemes, and Figure 12c shows the CDF of the *LE* under TDMA+FDMA transmission schemes. It is worth noting that the x-axis units are different in Figure 12b,c.

As shown in Figure 12b, the positioning performance decreased upon shortening the positioning period in the TDMA-only scheme. In the 1000 ms positioning period, 96% of the *LE* was below 20 cm, and 99% was below 50 cm. In the 600 ms positioning period, the positioning accuracy was slightly reduced; 93% of the *LE* was below 20 cm, and 99% was below 50 cm. When the positioning period was shortened to 450 ms, the positioning performance of the system was poor; only about 30% of the *LE* was less than 20 cm, and only about 33% was less than 50 cm. However, as shown in Figure 12c, in the TDMA+FDMA transmission scheme, the positioning performance was almost the same under the three different positioning periods; about 90% of the *LE* was less than 20 cm, and about 99% was less than 50 cm. Hence, the TDMA+FDMA transmission scheme was more powerful in realizing centimeter-level accuracy positioning under a high update rate.

### 3.3. Moving-Target Positioning Experiments 

**Figure 13:**
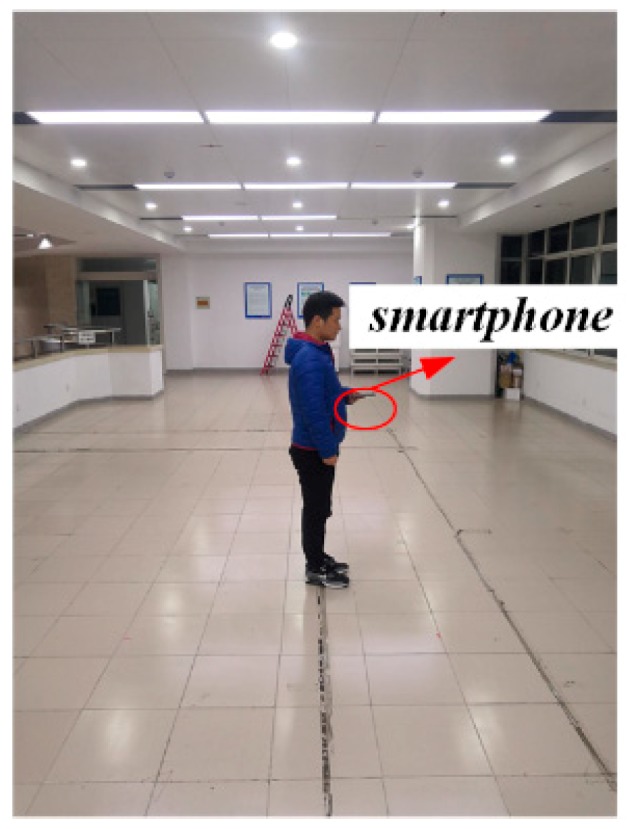
Diagram of smartphone’s position during experiment.

To demonstrate the feasibility and the validity of the proposed positioning correction scheme, moving-target positioning experiments were conducted using the TDMA+FDMA transmission scheme with a 1000 ms positioning period. Three tracks were planned in the positioning area. A user with the smartphone, as shown in Figure 13, moved on each track according to the direction specified by the black arrow at three different speeds, namely, slow-speed, medium-speed, and high-speed. The approximate ranges were 0.4–0.5 m/s for slow-speed, 0.6–0.7 m/s for medium-speed, and 0.8–0.9 m/s for high-speed. The user walked five laps on each track at each speed. 

**Figure 14:**
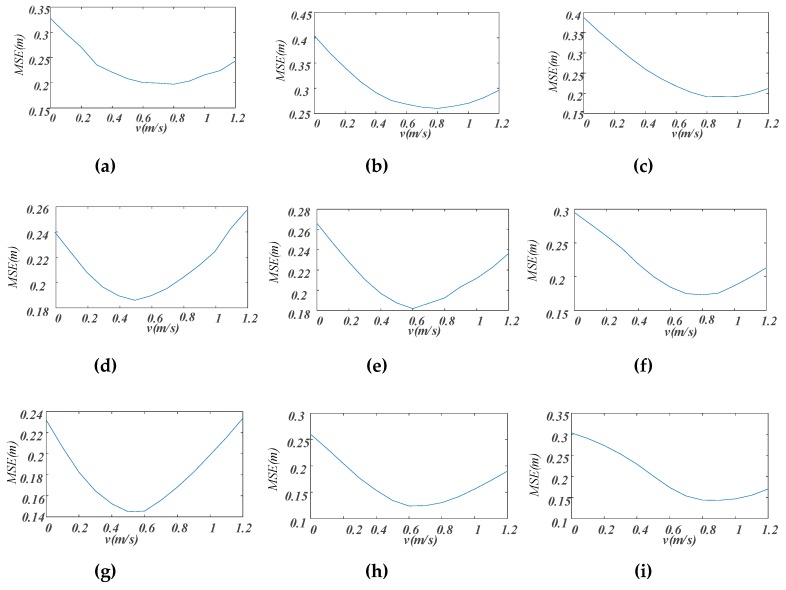
Relationship between positioning mean squared error (MSE) and *v* values. (**a**) Track 1 slow-speed; (**b**) Track 1 medium-speed; (**c**) Track 1 high-speed; (**d**) Track 2 slow-speed; (**e**) Track 2 medium-speed; (**f**) Track 2 high-speed; (**g**) Track 3 slow-speed; (**h**) Track 3 medium-speed; (**i**) Track 3 high-speed.

With the measured TDOA data, two kinds of positioning correction experiments were carried out. In the first experiment, the Doppler shifts were calculated at different fixed speeds but not at the estimated speed. In other words, the *v* in Equation (6) was not calculated according to Equation (9) but was set to fixed values in the range of 0–1.2 m/s at intervals of 0.1 m/s. As the target moving directions were determined in advance for each track, the θ at each positioning point was calculated according to Equation (11) directly. Figure 14 gives the relationship between the positioning MSE and *v* value for the three tracks and the three different speeds. When *v* = 0, the MSE was the average error without positioning correction. It was found that the greater the target moving speed was, the greater the MSE before positioning correction was. In all cases, the MSE decreased first and then increased as *v* increased, and the *v* value corresponding to the minimum MSE approximated the actual target moving speed. Generally, the optimal value of *v* for slow-speed was near 0.5 m/s, except for Track 1, and the optimal values of *v* for medium-speed and high-speed were near 0.6 and 0.8 m/s, respectively. This experimental result verifies that it is possible to obtain good positioning correction performance when the *v* is estimated from the actual speed of the moving target. 

**Figure 15:**
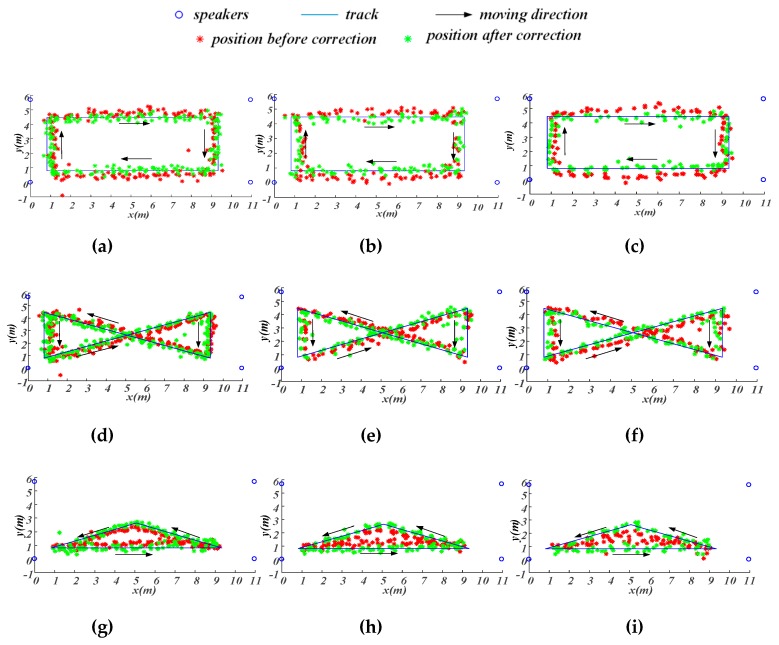
Results of moving-target positioning experiments. (**a**) Track 1 slow-speed; (**b**) Track 1 medium-speed; (**c**) Track 1 high-speed; (**d**) Track 2 slow-speed; (**e**) Track 2 medium-speed; (**f**) Track 2 high-speed; (**g**) Track 3 slow-speed; (**h**) Track 3 medium-speed; (**i**) Track 3 high-speed.

In the second positioning experiment, the Doppler shifts were calculated according to Equations (6)–(11) where 2 m/s was set as the speed threshold. When the speed estimated by Equation (9) was above 2 m/s, no TDOA update and positioning correction were made. Figure 15 demonstrates the positioning results for the three tracks under the three different speeds. The red points are the positions before correction and the green points represent positions after corrections. Besides, the blue lines are real tracks. For the sake of clear display, the results with positioning error above 50 cm were not presented in Figure 15. From Figure 15, it can be found that positioning results without correction were always on one side of the real track due to the influence of the Doppler shift for all the three tracks. After correction, the positioning results were closer to the real track and distributed on both sides of the real track. As a supplement, Table 3 gives the percentage of the abnormal positioning with errors greater than 50 cm. As can be seen from Table 3, TDOA correction based on Doppler shift could greatly reduce the proportion of results with positioning errors greater than 50 cm. Table 4 summarizes the MSE for all cases. The MSE values before correction are given on the left side, and those after correction are on the right side in parentheses. The data in Table 4 show that, after correction, the positioning errors were reduced by about 10 cm. 

## 4. Discussion

### 4.1. Feasibility and Superiority of TDMA+FDMA Transmission Scheme in Improving System Update Rate

The TDOA-chirp-based indoor positioning systems used in related studies generally adopted the TDMA-only transmission scheme to control chirp signals. For instance, Sérgio I. Lopes et al. designed a system that used four speakers to transmit chirp signals of 18–22 kHz frequency under the TDMA-only scheme [5]. In their research, the positioning period, defined as the time between the signal transmission and the position estimation, had a mean value of 350 ms, and 96% of the *LE* was below 20 cm in a positioning area of 9 m × 8 m. In this study, the positioning performance decreased upon shortening the positioning period in the TDMA-only scheme. When the positioning period was 1000 ms, 96% of the *LE* was below 20 cm, and 99% was below 50 cm. Considering the positioning accuracy and the update rate, it seems that our positioning accuracy is inferior to that in Sérgio I. Lopes et al.’s research. However, the different experimental environments make the results of these two studies incomparable. The experiments in Lopes et al.’s research were carried out in a regular room with concrete walls and ceiling, windows, linoleum floor, and different types of office furniture providing relatively less reflection. However, the experiment environment of our system was in the lobby of an office building with a great deal of noise and significant reflection. As mentioned in the introduction, the detection performance of chirp signals is influenced by the TBP, which is defined as the product of the duration of the signal and the bandwidth. Generally, the larger the TBP is, the higher the time resolution and the accuracy of chirp signal detection will be. In the research of Lopes et al., a 10 ms signal duration, 4 kHz bandwidth, and 40 kHz·ms TBP was used for localization. In our experiments, the minimum positioning period was 450 ms, in which the signal duration time, the bandwidth, and the TBP were 20 ms, 7 kHz, and 140 kHz·ms, respectively, and about 30% of the *LE* was less than 20 cm, and 33% was less than 50 cm. The results show that the experimental environment in our study puts forward higher requirements for TBP. When the positioning period is less than 450 ms, the positioning accuracy cannot be guaranteed. 

Although the combination of TDMA and FDMA has been widely used in related fields, such as wireless networks [36], microcellular systems [37], underwater acoustic networks [38], and mobile radio systems [39], it is the first attempt to realize the scheme in indoor localization systems. In FDMA, the 15–22 kHz frequency band was divided into two sub-bands: 18–15 kHz and 19–22 kHz. In TDMA, four nodes were divided into two groups to transmit the two sub-band chirp signals. The results of the static-target positioning experiments showed that the TDMA+FDMA transmission scheme has more advantages in improving the update rate than the TDMA-only scheme. The positioning performances were almost the same under three different positioning periods, and about 90% of the *LE* was less than 20 cm, and 99% was less than 50 cm, even for the 450 ms positioning period. For the 450 ms positioning period, the signal duration time, the guard time, the remaining time, and the TBP were 50 ms, 150 ms, 50 ms, and 150 kHz·ms, respectively. Thus, the TDMA+FDMA scheme had a higher TBP value than the TDMA-only scheme, which may have been one of the reasons why the former exhibited higher positioning accuracy. 

However, although the TDMA+FDMA scheme was verified to be an effective solution to improve the system update rate, as a preliminary work, this study failed to show the extent to which this scheme can shorten the positioning period. Since the frequency band of chirp signals in the TDMA+FDMA scheme was less than half of that in the TDMA-only scheme, the signal duration time was set to 50 ms to ensure the TBP value, and a 150 ms guard time was adopted. In future work, the optimal combination of the signal duration time, the protection time, and the remaining time will be explored to improve the system update rate further.

### 4.2. Feasibility and Superiority of Target-Moving-Speed-Based TDOA Correction Method in Improving Positioning Accuracy

It is generally known that, for a moving target, the signal received exhibits Doppler shifts, which can influence the performance of TDOA estimates [10,40]. The research on Doppler shift has mainly focused on outdoor positioning systems. For instance, Slamet Widodo et al. designed a GPS-based positioning system that used Doppler shift compensation for moving target positioning [41]. In their research, the Doppler shift was estimated by detecting the maximum value of the power spectrum and was then used to re-generate a new referred signal to estimate the arrival time. The results showed that, when the target moved in a circular path with an angular velocity of 0–1.3 rad/s, the positioning error before compensation was about 50 mm at 0.2 rad/s and 120 mm at 0.4 rad/s. When Doppler shift compensation was used, the target could be localized to within 25 mm of the actual position. However, there are very few studies on reducing the effect of the Doppler shift on the positioning accuracy of indoor positioning systems.

To the best of our knowledge, this study is the first to deal with Doppler frequency shift in acoustic indoor positioning systems. In particular, a target-moving-speed-based TDOA correction method was used to improve the positioning accuracy of a TDOA-chirp-based indoor positioning system. The basic idea of this method is to convert the Doppler shifts in the frequency domain into time differences to update the TDOA values. The results of moving-target positioning experiments verified that it is possible to obtain correction performance when the *v* is estimated from the actual speed of the moving target, and the positioning error was reduced by about 10 cm when the proposed method was adopted. In other words, the proposed target-moving-speed-based TDOA correction method can be considered as an effective method to reduce the positioning error caused by the Doppler shift.

However, although its effectiveness was verified, the proposed correction method is still very preliminary, and there are at least two problems that should be paid attention to and solved to improve its performance in the future.

Firstly, when the Doppler shift was calculated using Equation (6), the speed *v* of the moving target and the angle *θ* between the directions of target motion and audio signal propagation were estimated based on the previous position and the current position. Taking the 1000 ms positioning period and the speed of 0.6–0.7 m/s as an example, the position difference between the two positionings was about 60–70 cm. As shown in Table 4, the positioning errors ranged from 30–40 cm. These large positioning errors may have caused relatively large speed and direction estimation errors, thereby affecting the accuracy of estimation of the Doppler frequency shift. Therefore, more effective methods for the accurate estimation of Doppler frequency shifts caused by target motion should be explored. Accelerometers and gyroscopes can be considered for accurate detection of the target moving speed and direction.

Secondly, the Doppler shift ∆f was converted into the time difference ∆t using Equation (7) based on the assumption that the linear chirp signal with Doppler shift was still a linear chirp signal, and the TDOA correction based on Doppler shifts was carried out according to Equation (8). However, the assumption that the received signal was still a linear chirp signal was made for the convenience of calculation. In fact, it is not very rigorous. Usage of the Doppler frequency shift to achieve more accurate TDOA correction is of great significance to improving the positioning accuracy for moving targets.

## 5. Conclusions

Taking an indoor positioning system based on audio chirp signals and TDOA as the research target, we proposed a TDMA+FDMA signal transmission scheme to improve the positioning update rate and a target-moving-speed-based TDOA correction method to reduce the positioning error caused by Doppler frequency shift. The feasibility and the superiority of the proposed methods were verified in static-target positioning and moving-target positioning experiments. This research provides a possible framework for the realization of a TDOA-chirp-based audio indoor positioning system with a high positioning accuracy and update rate.

## Figures and Tables

**Table 1:** Specific parameter values of three TDMA+FDMA transmission schemes.

	Period (ms)	Signal Duration (ms)	Guard Time (ms)	Remaining Time (ms)
**Scheme 1**	1000	50	150	600
**Scheme 2**	600	50	150	200
**Scheme 3**	450	50	150	50

**Table 2:** Specific parameter values of three TDMA-only transmission schemes.

	Period (ms)	Signal Duration (ms)	Guard Time (ms)	Remaining Time (ms)
**Scheme 1**	1000	50	150	200
**Scheme 2**	600	20	100	120
**Scheme 3**	450	20	70	90

**Table 3:** The percentage (%) of the abnormal positioning with errors greater than 50 cm.

	Track 1 (before/after)	Track 2 (before/after)	Track 3 (before/after)
**Slow-speed**	15.20/7.35	7.17/5.02	3.77/3.14
**Medium-speed**	29.11/14.56	9.44/6.67	7.14/0
**High-speed**	25.87/4.90	10.14/4.05	17.17/0

**Table 4:** MSE (cm) of moving-target positioning experiments.

	Track 1 (before/after)	Track 2 (before/after)	Track 3 (before/after)
**Slow-speed**	32.72/20.02	23.99/18.80	23.21/15.02
**Medium-speed**	40.43/27.03	26.62/18.49	26.02/12.03
**High-speed**	38.76/18.21	29.52/16.57	30.37/13.85
